# How to achieve an optimal alignment in medial opening wedge high tibial osteotomy?

**DOI:** 10.1186/s43019-021-00130-2

**Published:** 2022-02-08

**Authors:** Byoung Youl Kang, Do Kyung Lee, Hyeon Soo Kim, Joon Ho Wang

**Affiliations:** 1 Department of Orthopaedic Surgery, Sungkyunkwan University School of Medicine, Samsung Medical Center, Seoul, Republic of Korea; 2 Department of Orthopaedic Surgery, Samsung Changwon Hospital, Sungkyunkwan University School of Medicine, Changwon, Republic of Korea

**Keywords:** Medial opening wedge high tibial osteotomy, High tibial osteotomy, Undercorrection, Overcorrection, Optimal alignment, Operative planning, Correction error, Mechanical axis, Soft tissue laxity

## Abstract

Medial opening wedge high tibial osteotomy (MOWHTO) is a widely used surgical treatment option for medial compartmental osteoarthritis with varus deformity. It is important that proper lower limb alignment is achieved. However, there has been no consensus about an optimal alignment in MOWHTO. Most studies suggest that achieving valgus alignment is necessary, and recent studies support slight valgus mechanical alignment of less than 3° of mechanical femorotibial angle. Overcorrection and undercorrection is not recommended for achieving good surgical outcomes. To prevent undercorrection and overcorrection in MOWHTO, the method of placing the weight-bearing line in the target range must be precise. There are several ways to place a weight-bearing line within the target range. While the most important factor for a successful MOWHTO is achieving an ideal mechanical axis correction, there are a few other factors to consider, including joint line obliquity, posterior tibial slope, ligament balancing, and patellar height. Several factors exist that lead to undercorrection and overcorrection. Preoperative amount of varus deformity, lateral hinge fracture, and fixation failure can result in undercorrection, while medial soft tissue laxity and the amount of correction angle and target point beyond hypomochlion can result in overcorrection. This study aimed to review the literature on optimal alignment in MOWHTO and report on the factors to be considered to prevent correction errors and how to achieve an optimal alignment.

## Introduction

Medial opening wedge high tibial osteotomy (MOWHTO) is a widely used surgical treatment option for medial compartmental osteoarthritis with varus deformity in relatively young and active patients [1–4]. The mechanism of MOWHTO involves correcting the weight-bearing axis to reduce excessive load on the medial compartment and shifting it to the lateral compartment [5]. The goals of this approach are to reduce pain, improve the patient’s activity level, and delay the progression of osteoarthritis to decrease the need for knee replacement arthroplasty [6]. Many studies have reported that achieving proper lower limb alignment in MOWHTO is an important factor leading to good clinical outcomes [7–9].

Although numerous surgical techniques have been proposed, unintended correction errors still occur after MOWHTO [10–12]. Several studies have reported that optimal correction of the mechanical axis is difficult to achieve and only 70–80% of the postoperative mechanical axis is placed within the targeted range [13, 14]. Even though the bony correction is as accurate as planned preoperatively, correction errors of the mechanical axis still occur [11].

This study aimed to review the literature on an optimal alignment in MOWHTO and report on the factors to be considered to prevent correction errors and how to achieve an optimal alignment.

What is an optimal alignment in MOWHTO?

There has been no consensus about an optimal alignment in MOWHTO, and most studies suggest that achieving valgus alignment is necessary to acquire long-term clinical success and prevent recurrence of varus deformity [15]. Fujisawa et al. [16] reported good results when the postoperative weight-bearing line passed 30–40% laterally from the center of the knee joint. In postoperative arthroscopy, cartilage remodeling by fibrous cartilage was found in patients with properly performed high tibial osteotomy (HTO). Coventry et al. [17, 18] suggested an alignment of 10° valgus of the anatomical femorotibial angle [3–5° of mechanical femorotibial angle (mFTA)] is optimal. With respect to longevity, 3–5° of mFTA showed excellent outcomes at 10 years of follow-up. Similarly, Hernigou et al. [19] reported that among 93 knees treated with MOWHTO, 22 knees with 3–5° of mFTA obtained the best results after an average follow-up of 11.5 years, in terms of pain relief and prevention of joint arthrosis progression.

Recent studies have different opinions from studies that emphasized valgus alignment, which is more than 3° of valgus mFTA. In a recent biomechanical study [20], beyond 3° of valgus mFTA there is no benefit in terms of reducing the pressure on the medial compartment without damage to the lateral compartment cartilage. In a computer-simulated knee model study [21], the peak contact pressure of the medial compartment when walking significantly decreased in only neutral mechanical alignment, and it was lower than peak contact pressure of the lateral compartment. The same results were obtained when squatting. Similarly, Atkinson et al. [22] reported that valgus alignment is not necessary, based on the finding that correction to near neutral alignment rather than excessive valgus alignment is sufficient to provide regenerative stimulation to the articular cartilage of the medial compartment without damaging the lateral compartment. Although there are inherent limitations in biomechanical studies, the findings of which may differ from the clinical outcomes of actual patients, these results need to be considered.

Jakob et al. [23] suggested that the target mechanical axis should not be absolute, but should be considered according to each patient’s articular cartilage state as MOWHTO induces regeneration of damaged cartilage. They recommended that the correction angle should be adjusted according to the residual thickness of the cartilage in the medial compartment. The mechanical axis should pass 10–15% laterally from the center of the tibial plateau when one-third of the medial cartilage is damaged, 20–25% when two-thirds is damaged, and 30–35% when full thickness of cartilage is impaired. Based on this perspective of cartilage regeneration, Kim et al. [24] performed concomitant cartilage procedures (microfracture, autologous chondrocyte implantation, and stem cell implantation) with MOWHTO and reported good clinical outcomes in cases with mechanical axis of 0–3° valgus.

Most studies recommended avoiding undercorrection (varus alignment) or excessive overcorrection to obtain good clinical outcomes [16, 25]. Undercorrection is considered as treatment failure, leading to subsequent progression of medial compartment osteoarthritis, persistent pain, and poor clinical outcomes [19]. El-Azab et al. [26] reported that the Lysholm score was lower at 3, 6, and 36 months follow-up in the undercorrection group, in which the weight-bearing line passed under 50% from the medial border of the tibial plateau after MOWHTO, compared with that in the proper correction group. Moreover, clinical outcomes inferior to that of the proper correction group were observed in the undercorrection group. Furthermore, undercorrection in MOWHTO may require next-stage procedures, such as total knee replacement arthroplasty [27].

In addition, overcorrection leads to unsatisfactory clinical outcomes. Although there is no clear determination for overcorrection, it could result in cosmetic problems that cause patients to feel dissatisfied and lead to poor clinical outcomes [28]. Excessive loading in the lateral compartment can also result in lateral compartmental arthritis [29]. Furthermore, the overcorrected valgus knee could increase patellofemoral contact pressure, leading to patellofemoral pain and degenerative changes [30]. Lee et al. [31] reported inferior clinical outcomes in patients with overcorrection due to patellofemoral maltracking and patellofemoral pain. Moreover, in cases of overcorrected valgus knees, there may be technical difficulties in ligament balancing during conversion total knee replacement arthroplasty [32].

## Perioperative planning

To prevent undercorrection and overcorrection in MOWHTO, the method of placing the weight-bearing line in the target range must be precise and detail oriented. There are several ways to place a weight-bearing line within the target range. These methods are classified into conventional and navigation-assisted methods, and conventional methods are further classified into preoperative and intraoperative methods according to the timing of calculation of the correction angle.

### Conventional methods

#### Preoperative methods

Preoperative methods involve calculating the correction angle using a weight-bearing scanogram with calibration before operations.

##### Miniaci’s method [33]

**Fig. 1:**
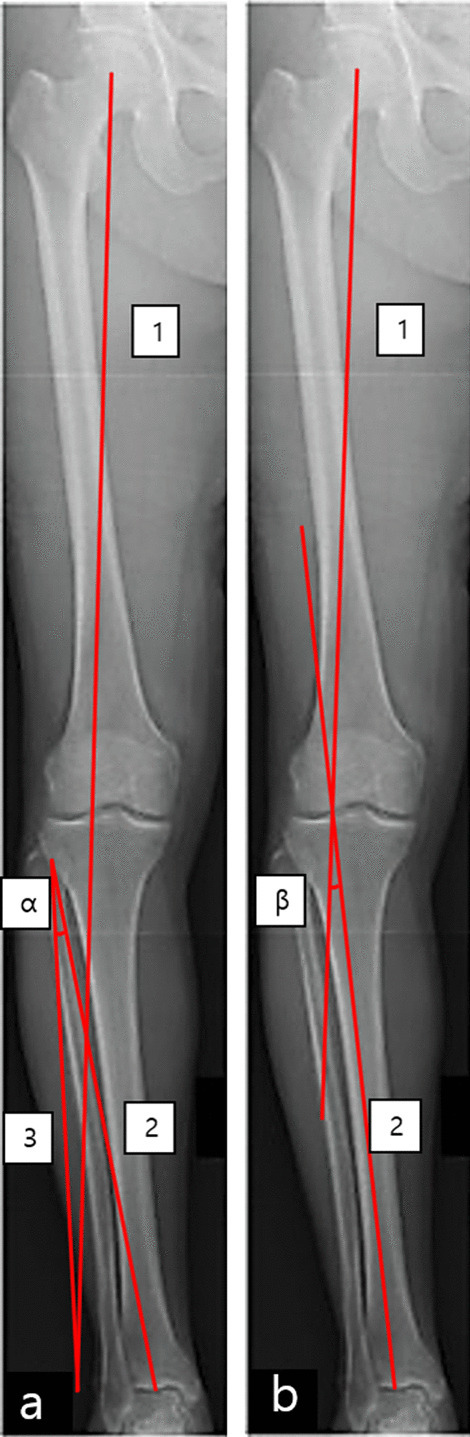
Miniaci’s method and Dugdale’s method. **a** Miniaci’s method: Line 1 is the planned weight-bearing line. The angle between lines 2 and 3 is the planned correction angle α. **b** Dugdale’s method: Line 1 runs from the center of the hip joint to the target point of the tibial plateau. Line 2 runs from the center of the ankle joint to the target point of the tibial plateau. The angle between lines 1 and 2 is the planned correction angle β

Line 1 is a planned weight-bearing line that runs from the center of the hip joint to the predicted new center of the ankle joint and passes through the predetermined target point of the tibial plateau. Line 2 runs from the hinge point of the osteotomy to the center of the ankle joint. Line 3 runs from the hinge point of the osteotomy to the predicted new center of the ankle joint. The angle between lines 2 and 3 is the planned correction angle α (Fig. 1a). Lee et al. [34] reported that this method showed high inter- and intra-rater reliabilities in the preoperative correction angle and osteotomy gap. Elson et al. [35] reported that Miniaci’s method is reliable regardless of an observer’s experience.

##### Dugdale’s method [36]

Line 1 runs from the center of the hip joint to the predetermined target point of the tibial plateau (Dugdale suggested 62% of the tibia plateau from the medial border). Line 2 runs from the center of the ankle joint to the predetermined target point of the tibial plateau. The angle between lines 1 and 2 is the planned correction angle β (Fig. 1b). Schröter et al. [37] reported that the difference between the planned correction angle and postoperative corrected angle in MOWHTO using Dugdale’s method was 0.8°. Blackburn et al. [38] reported that Dugdale’s method showed high inter- and intra-rater reliabilities, which were not different from those of Miniaci’s method.

##### Real-size weight-bearing scanogram method

**Fig. 2:**
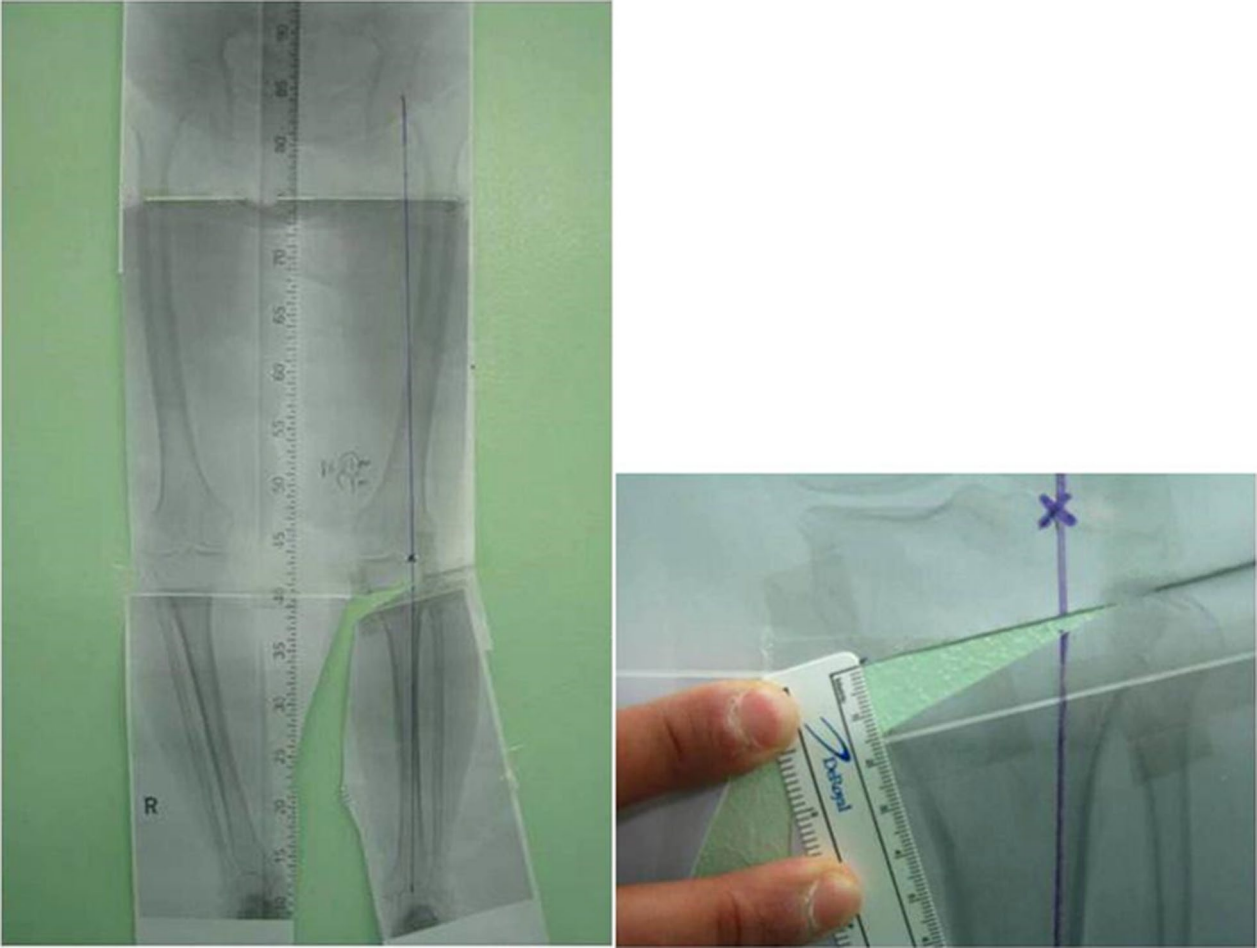
Real-size weight-bearing scanogram method. With a full-size radiograph printed on paper, a line is cut along where the osteotomy is going to be performed. The tibia is rotated until the weight-bearing line passed through the target point. Reprinted with permission from Lee DH: The weight-bearing scanogram technique provides better coronal limb alignment than does the navigation technique in open high tibial osteotomy. The Knee. Copyright © 2012 Elsevier B.V. All rights reserved

Lee et al. [39] reported that measurement errors could occur due to magnification differences on computer screens in methods using a picture archiving and communication system. To compensate for this problem, they suggested a method to plan the correction angle and osteotomy gap by taking a weight-bearing scanogram and printing it in actual size (100%). With a full-size radiograph printed on paper, the aiming point is marked on the tibial plateau. Second, using scissors, a line along which the osteotomy is to be performed is cut. Third, the distal part of the paper is rotated from its hinge to make a straight line from the center of the hip joint, passing through the target point of the tibial plateau, to the center of the ankle joint. Then, the gap and angle of the wedge are measured (Fig. 2). Using this method, high accuracy and low outliers were reported in the postoperative mechanical axis passing within the target range [39]. However, despite its high accuracy, it requires a large size of paper and takes a long time, which is a disadvantage of this method.

##### Three-dimensional printing patient-specific instrument

As in other areas of orthopedic surgery, in MOWHTO, patient-specific instrument(PSI) was introduced, paying attention to the differences in the anatomical structures of each patient. After making a simulative bone through 3-dimensional printing by taking a computed tomography, the manufacturer makes the instruments, and the surgeon performs the operation using the instruments. Yang et al. [40] obtained postoperative mechanical axis close to preoperative planning without significant change in posterior tibial slope (PTS) with PSI. In contrast, when comparing conventional, navigation, and PSI, although PSI showed better precision, there was no statistically significant difference [41].

#### Intraoperative methods

##### Cable or rod method

**Fig. 3:**
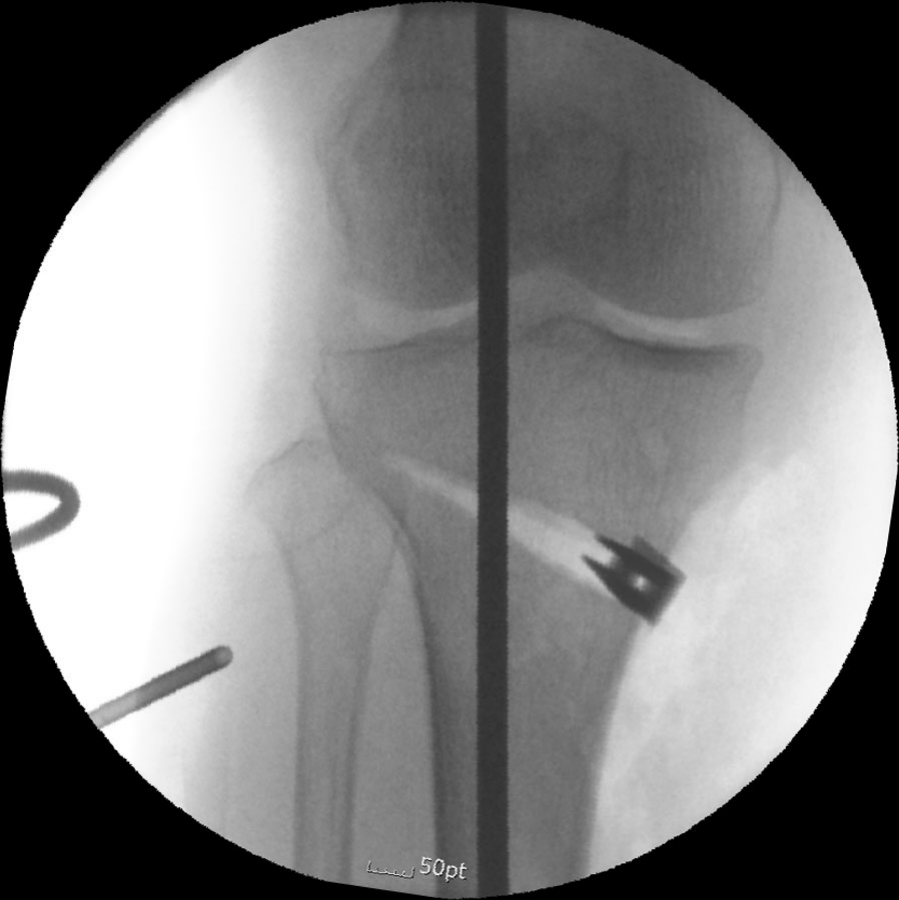
Intraoperative method under fluoroscopy guidance with a Bovie cable or radiopaque rod

An intraoperative method under the guidance of fluoroscopy with a Bovie cable or radiopaque rod is widely used because it is easy and allows real-time monitoring (Fig. 3). However, in one study, this method resulted in identification of a higher correction error than did preoperative planning methods, and tended to cause undercorrection [42]. In contrast, in a recent study, this method tended to cause overcorrection, and the accuracy was inferior to the preoperative Miniaci method [43]. This can be attributed to the non-weight-bearing state. In this regard, Kim et al. [44] proposed performing a valgus stress test intraoperatively to reproduce the weight-bearing state. With this procedure, they achieved a postoperative mechanical axis within an acceptable range with reduced outliers.

### Navigation-assisted method

Recently, navigation systems have been introduced for MOWHTO. Several meta-analysis review studies [45–47] reported that the navigation-assisted method increased accuracy by reducing outliers and was superior to the conventional methods in terms of reliability and reproducibility. This could be attributed to the real-time feedback of the mechanical axis when an operator conducts an osteotomy. In addition, it was reported that the navigation-assisted method was significantly advantageous in terms of preserving the PTS [46]. A three-dimensional analysis would enable better maintenance of PTS than the conventional method would.

Despite these advantages, it has several disadvantages. Navigation-assisted MOWHTO requires additional operation time compared with conventional surgery, and there is an additional cost of purchasing a navigation device. Moreover, an infection may occur through a stab wound made by the pin used for the navigation system [48]. Nha et al. [47] reported that superior accuracy in the mechanical axis correction was not clearly linked to statistically better clinical outcomes. The navigation-assisted method inherently does not reflect the weight-bearing state; therefore, there could be a correction error resulting from the difference between the supine and standing positions. In this regard, Kyung et al. [11] reported that the postoperative mechanical axis tended to be overcorrected by a mean of 2° from the planned mFTA, although the amount of bony correction was appropriate.

Among the various planning methods, it is difficult to conclude which method is superior. However, it should be noted that when a surgeon selects an intraoperative method (cable method or navigation-assisted method), the alignment can change after the operation because they do not reflect the weight-bearing state.

## Other considerations in preoperative planning

While the most important factor for a successful MOWHTO is achieving an optimal mechanical axis correction, there are a few other factors to consider (Table 1). In genu varum, the factor due to the tibia accounts for only 30% [49], correction of the proximal tibia alone, such as with MOWHTO, can lead to nonphysiologic morphological characteristics such as joint line obliquity (JLO) [50]. In a study using a three-dimensional finite element model, Nakayama et al. [51] reported that the shear stress of tibial articular cartilage significantly increased when JLO of more than 5° occurred, and Song et al. [52] reported the Knee Society objective and functional scores, used to evaluate clinical outcomes, were significantly low in patients with JLO greater than 4°. In terms of medial proximal tibial angle (MPTA), an important radiological parameter for anticipating postoperative JLO, Nakayama et al. [51] suggested that 95° of MPTA corresponds to 5° of JLO. Oh et al. [53] reported that patients with severe varus deformity requiring a large amount of correction tended to have JLO greater than 4°. Therefore, if the anticipated MPTA is more than 95° or JLO is more than 4° or 5°, another option such as double-level osteotomy of the femur and tibia should be considered rather than MOWHTO.

**Table 1:** Factors that should be considered in preoperative planning of medial opening wedge high tibial osteotomy

•Determination of the target mechanical angle
•Selection of the planning method
•Joint line obliquity
•Posterior tibial slope
•Ligament insufficiency (anterior or posterior cruciate ligament)
•Patellar height (anterior knee pain or patellofemoral arthritis)

**Fig. 4:**
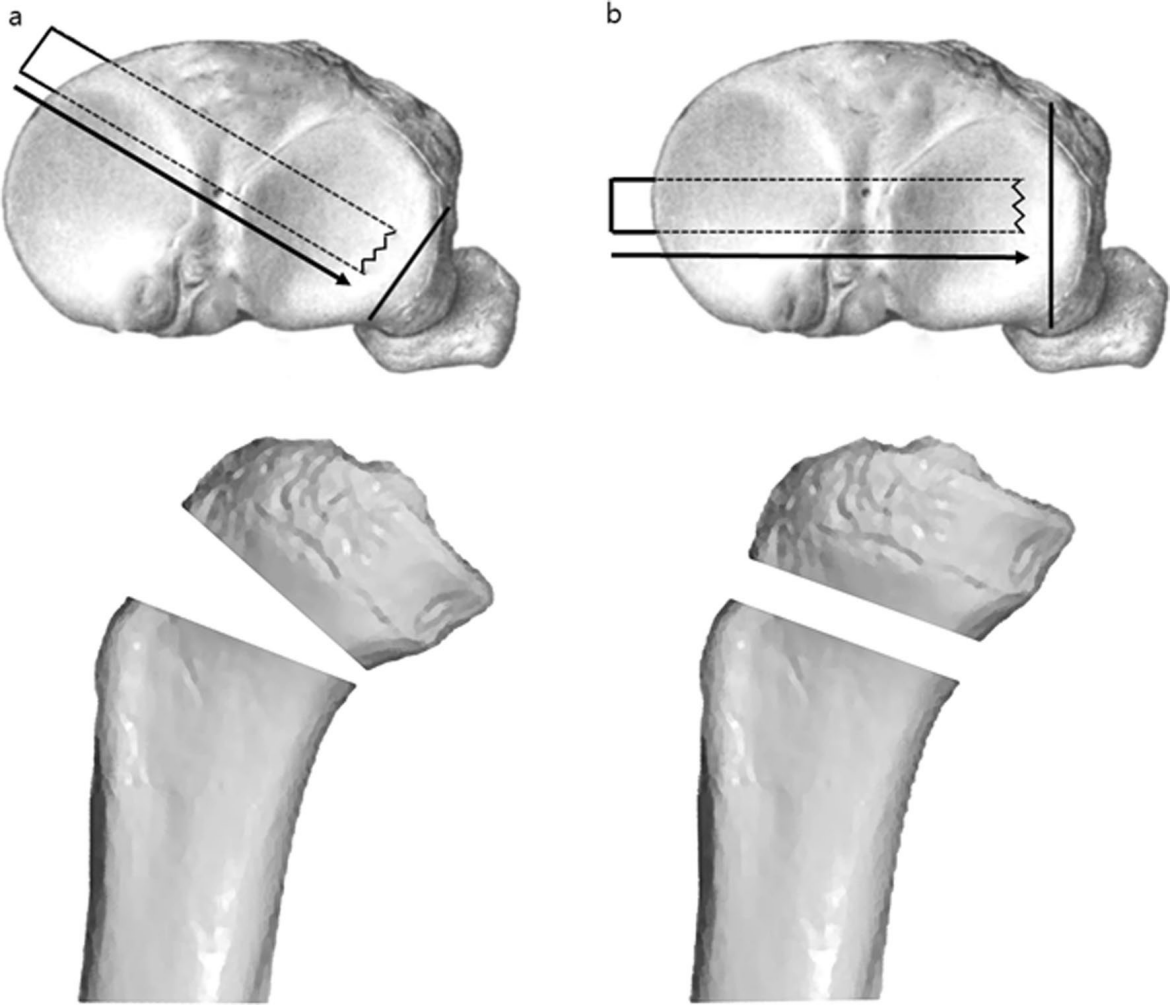
The effect of the cortical hinge on the posterior tibial slope. **a** Posterolateral cortical hinge osteotomy with uneven anterior and posterior gap. **b** True lateral cortical hinge osteotomy with even anterior and posterior gap

The purpose of HTO is to correct the mechanical axis in the coronal plane, but it unintentionally causes a change in the sagittal plane as well. El-Azab et al. [54] performed HTO in 120 patients, of whom 60 underwent MOWHTO and 60 underwent lateral closing wedge high tibial osteotomy (LCWHTO). PTS increased in all cases of MOWHTO and decreased in all cases of LCWHTO. In a meta-analysis, Nha et al. [55] found that the average PTS increased by approximately 2° in MOWHTO and decreased by approximately 2° in LCWHTO. According to Giffin et al. [56], an increase in PTS of 4.4° resulted in an anterior tibial translation of approximately 2° when an axial load of 200 N was applied, while the force applied to the posterior cruciate ligament (PCL) decreased. They explained that this phenomenon is due to the anteriorly directed force on the tibia generated by PTS, which is not perpendicular to the axial load. In this regard, for patients with PCL deficiency, MOWHTO would be advantageous as it reduces the force applied to the PCL. In contrast, in patients with anterior cruciate ligament deficiency, it would be disadvantageous because force applied to the anterior cruciate ligament increases. To maintain PTS, Song et al. [57] reported that the anterior opening gap should be 67% of the posterior opening. Wang et al. [58] pointed out that the true lateral side hinged opening cannot change the sagittal plane, but if MOWHTO is performed in the posterolateral hinge, it could cause changes in the sagittal plane. The posterolateral hinge causes a mismatch between the anterior and posterior gaps, leading to an increase in PTS. Therefore, they suggested that accurate lateral hinged osteotomy could maintain PTS within a normal range (Fig. 4).

MOWHTO inadvertently causes a change in the position of the patella. In a meta-analysis conducted by Bin et al. [30], when MOWHTO was performed, patellar height decreased by an average of 7%. This is because the distance between the knee joint line and tibial tuberosity is lengthened due to the position of the osteotomy proximal to the tibial tuberosity, which is the site where the patellar tendon is inserted [59]. The patella baja caused by the distally transferred patella increases the contact pressure of the patellofemoral joint, causing a degenerative change in the patellofemoral joint and resulting in anterior knee pain and limit of motion [60]. In the overcorrected valgus knee, Q-angle alteration is inevitable and increases the lateral pull on the patella, causing patellar maltracking during knee flexion [31, 59]. Kim et al. [61] reported that the International Cartilage Repair Society grade deteriorated in both the patellar and femoral cartilages in an arthroscopic second look conducted approximately 2 years after MOWHTO. Gaasbeek et al. [62] reported on a novel technique using biplanar distal tuberosity osteotomy, which opens the gap distally rather than proximally to the tibial tuberosity. Using this method of maintaining patellar height, Horikawa et al. [63] reported that patellar height was almost the same preoperatively and postoperatively.

## Which factors cause correction errors and how to avoid them?

Several factors exist that lead to undercorrection and overcorrection, and avoiding them is closely related to the success of MOWHTO, as mentioned earlier (Table 2).

**Table 2:** Pitfalls to to avoid correction error

	Factors	Pitfalls to avoid
Undercorrection	Preoperative amount of varus deformity Lateral hinge fracture Fixation failure (insufficient plate fixation)	Placing the lateral hinge at “Safe zone” Fixation with locking plate
Overcorrection	Severity of varus deformity (the amount of correction angle) Latent medial soft tissue laxity Intraoperative releasing of superficial medial collateral ligament	Taking preoperative valgus stress X-ray and adjusting latent medial laxity from target correction angle Using hypomochlion (valgus 2° of mFTA) as the target point

### Undercorrection

The occurrence of undercorrection is related to the preoperative amount of varus deformity. The more severe the varus, the more likely is occurrence of undercorrection. Kamada et al. [64] divided patients based on varus deformity greater than 5° and less than 5° and performed MOWHTO. They found that the group with greater than 5° varus deformity had a higher frequency of undercorrection and significantly lower postoperative Lysholm score than did the group with less than 5° of varus deformity. They hypothesized that these findings were a result of the more severe varus deformity causing more tightness of the medial side soft tissue.

Depending on the type of plate to be fixed after osteotomy, undercorrection or correction loss may occur. Given that the osteotomy gap must support body weight, it is affected by the stability of the plate fixation. Therefore, stable fixation is an important factor in maintaining the osteotomy gap. Hernigou et al. [65] reported that there was a lower correction loss rate and better clinical outcomes in patients treated with locking plates than in patients treated with conventional plates.

**Fig. 5:**
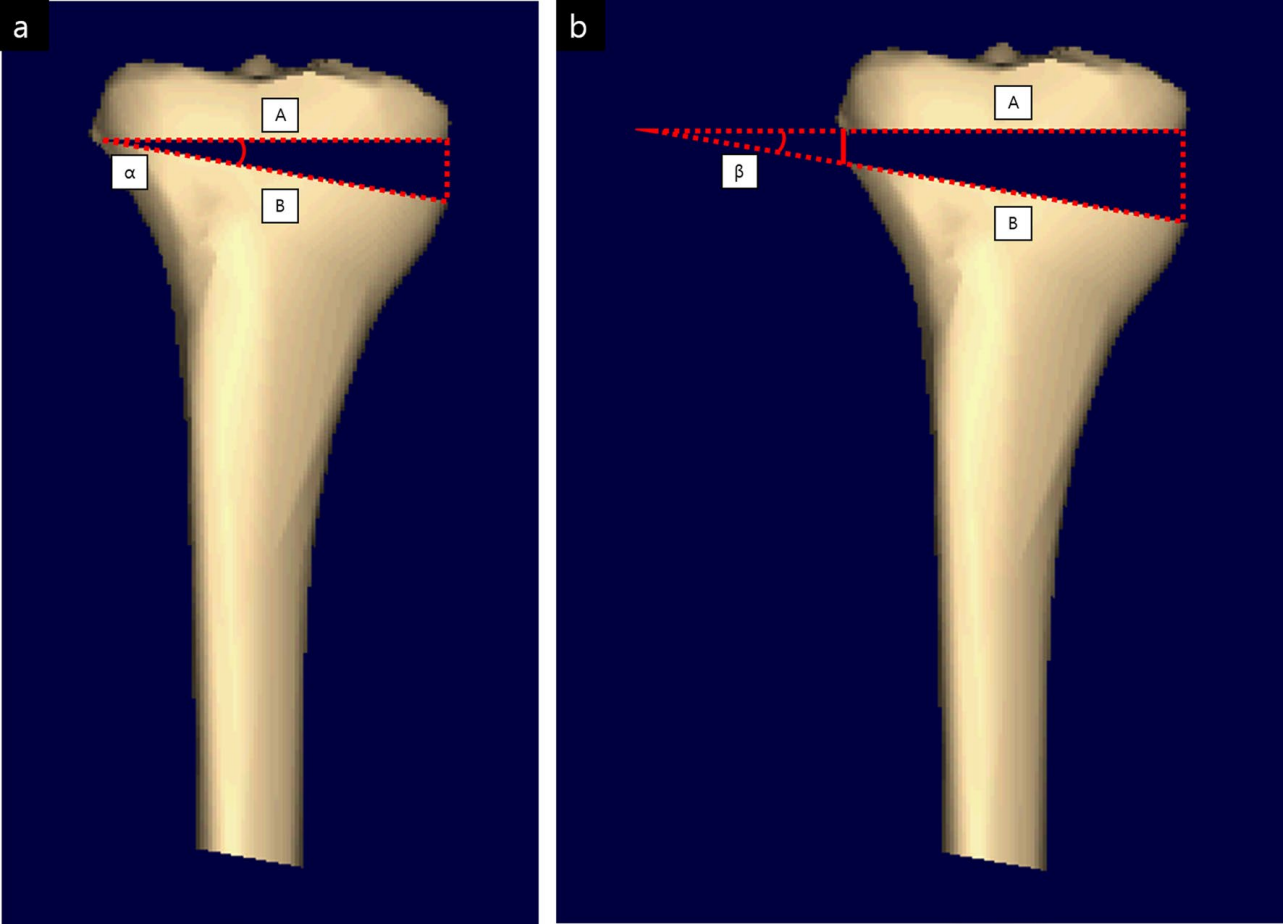
Correction error due to hinge fracture. **a** Properly conducted osteotomy and **b** Lateral hinge fracture. Correction error might be affected by the lateral hinge fracture

Lateral hinge fractures can seriously affect the maintenance of the osteotomy gap and lead to delayed union or nonunion and correction loss [66, 67]. Assuming that the preoperatively planned angle is α, the shape of the osteotomy at the hinge fracture becomes trapezoidal rather than triangular. Therefore, due to the elongated osteotomy upper and lower lines (A and B), the newly formed angle β becomes smaller than α, resulting in undercorrection (Fig. 5). In terms of risk factors for lateral hinge fracture, Lee et al. [68] reported that an increased medial opening gap increases the incidence of lateral hinge fractures. In their study, the lateral hinge fracture group had an opening gap of more than 12 mm. In cases requiring gaps of more than 12 mm, care should be taken. To prevent lateral hinge fractures, Nakamura et al. [69] and Han et al. [70] recommended a “safe zone” of hinge that is located in specific region following geographic relation to the proximal tibiofibular joint.

### Overcorrection

**Fig. 6:**
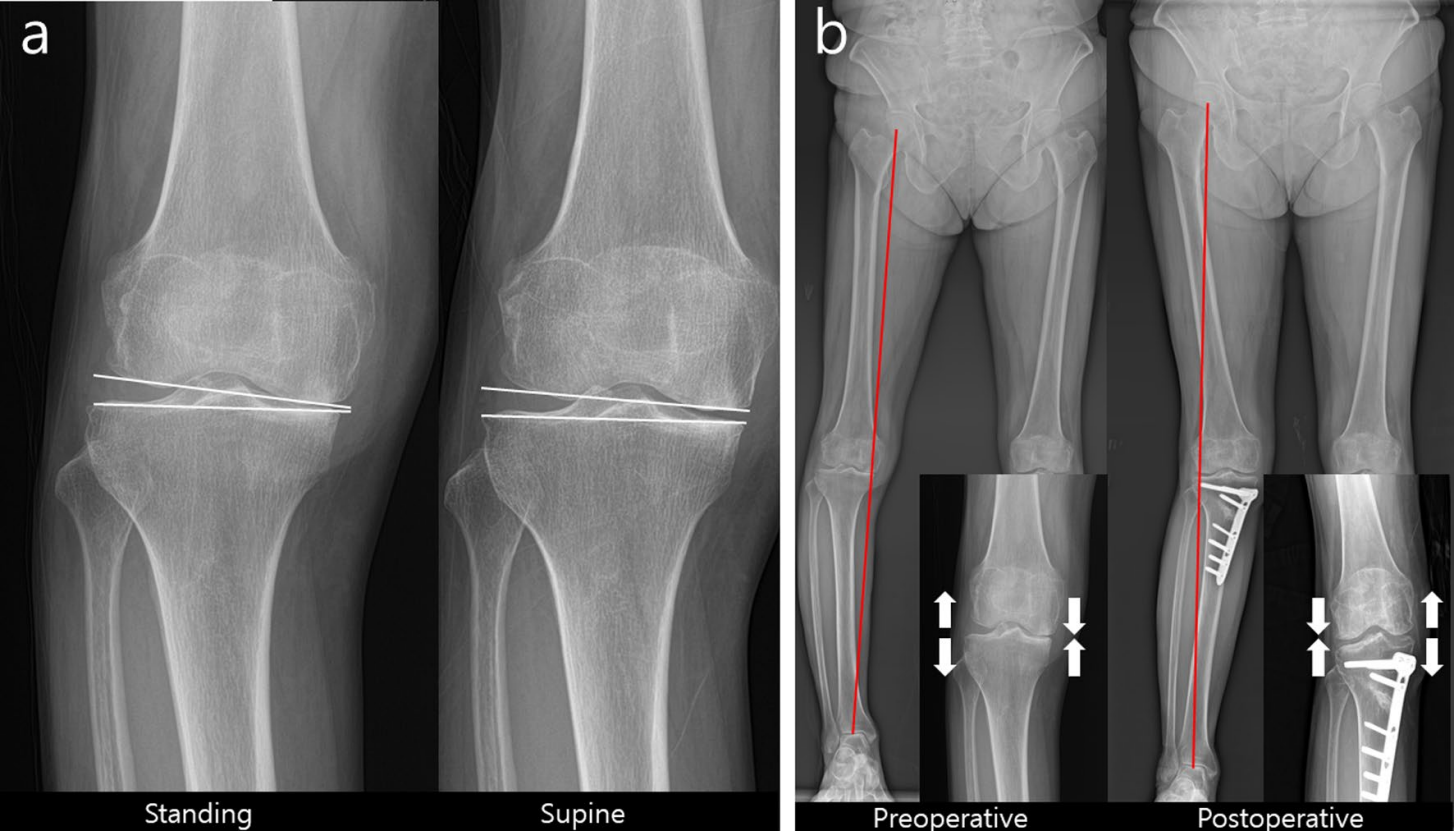
Perioperative change in the soft tissue tension around the knee. **a** Preoperative standing and supine knee radiographs. **b** Preoperative and postoperative scanogram. The difference of joint line convergence angle between weight-bearing and supine X-ray implies the possibility of soft tissue tension change after medial opening wedge high tibial osteotomy. The medial joint space is opened, and the lateral joint space is closed by shifting the weight-bearing axis after medial opening wedge high tibial osteotomy. This results in unexpected overcorrection

Recently, researchers have been interested in the discrepancy between the planned mechanical axis and the actual postoperative mechanical axis. For navigation-assisted MOWHTO, which showed better accuracy in bony correction than did conventional MOWHTO, some studies [11, 71] reported that the MPTA was corrected as planned, but the postoperative mechanical axis measured by a standing radiograph was overcorrected. They demonstrated that the inherent preoperative soft tissue laxity could be attributable for the difference between the preoperative and postoperative mechanical axes. Ogawa et al. [72] explained that the shift of the weight-bearing line after bony correction in MOWHTO changes the soft tissue tension around the knee (Fig. 6). Pape et al. [73] reported that intraoperative release of the superficial medial collateral ligament in MOWHTO, which cannot be quantified, could contribute to the tension around the medial soft tissue. Lee et al. [10] investigated the parameters that were correlated with overcorrection, and they found that a perioperative change in the joint line convergence angle (JLCA), which reflects soft tissue laxity, on the coronal plane is related to overcorrection. Lee et al. [74] attempted to identify the factors that affect the perioperative change in JLCA. In their study, latent medial soft tissue laxity, identified by a preoperative valgus stress radiograph, was statistically crucial to the perioperative change in JLCA. Moreover, a larger correction angle would cause a greater change in JLCA. In summary, these two factors, medial soft tissue laxity and severity of varus deformity, can result in overcorrection. On the basis of this concept, Ryu et al. [75]. suggested an equation adjusting latent medial laxity from the target correction angle. They targeted postoperative correction angle as valgus 3° of mFTA and subtracted one-third of delta JLCA, which means the JLCA on weight-bearing standing radiographs minus the JLCA on valgus stress radiographs(adjusted correction angle = target correction angle−1/3ΔJLCA). Heijens et al. [76] hypothesized that there is a certain mFTA beyond which JLCA changes significantly. They called it coronal “hypomochlion” and demonstrated that it is valgus 2° of mFTA (equivalent to the point where the mechanical axis passes through 57.5% of the tibial plateau from the medial border). In patients with considerable medial soft tissue laxity and severe varus deformity requiring a large correction angle, causing additional changes in JLCA, using hypomochlion (valgus 2° of mFTA) as the target point, can help prevent overcorrection.

## Conclusions

For successful MOWHTO outcomes, it is important to achieve an optimal alignment through accurate preoperative surgical planning. In addition, it is essential to recognize and pay attention to the correction amount, medial soft tissue laxity, risk factors for lateral hinge fracture, and other factors that can lead to correction errors. Moreover, the characteristics of each patient, such as JLO, PTS, ligament insufficiency, cartilage state, and patellofemoral degeneration, should be considered.

## Data Availability

Not applicable.

## Abbreviations

MOWHTO: Medial opening wedge high tibial osteotomy
HTO: High tibial osteotomy
mFTA: Mechanical femorotibial angle
PSI: Patient-specific instrument
PTS: Posterior tibial slope
JLO: Joint line obliquity
MPTA: Medial proximal tibial angle
LCWHTO: Lateral closing wedge high tibial osteotomy
JLCA: Joint line convergence angle
